# A Protest

**Published:** 1890-05

**Authors:** T. F. Allen

**Affiliations:** 10 East 36th Street, New York


### A PROTEST.

10 East 36th Street, New York, March 31st, 1890.

Editors of The Homœopathic Physician.

Gentlemen :—I find myself compelled to write to you complaining of the unfair treatment your journal pursues toward me personally. It is extremely distasteful for me to do this, but, since for twenty-five years and more I have faithfully and conscientiously practiced and taught straight Homœopathy as I understand it, and have persistently, in season and out of season, antagonized mongrelism in all its forms, it is not quite fair to be attacked in the way in which your journal is used for that purpose. It would give me great pleasure at any time to answer *in the same number of the journal* any article reflecting upon my professional character.

Concerning my definition of a homœopathic physician, I have simply to say that it is unassailable. Dr. P. P. Wells has seen fit in the last number of your journal to refer to me as ignorant. This imputation is just. Probably no one has more critically or more steadily pursued the study of our materia medica for twenty-five years than I have, and to-day I am profoundly impressed with my own ignorance of it, and I find, which I am sorry to say, is not found by some others, that my charity toward the opinions of others increases as my years increase. Dr. Wells chooses to refer as a parallel case to those who profess Christianity, and I think his reference is judicious. We call every one a Christian who professes himself as such and publicly unites with those professing a similar faith, and yet it is probable that no one lives a perfectly Christian life. The professing Christian does the best that he can, and in so doing is entitled to be called a professing Christian.

The rule of our Homœopathic Society in New York compels each one who unites with it to declare that he believes Homœopathy to be the best way of treating the sick; and when a man publicly unites with the Society and signifies his assent to this proposition he publicly declares himself a homœopathist and does the best he can to practice Homœopathy, and therefore he must be known to the public as a homœopathic physician. Some practice Homœopathy more, some less, each according to his knowledge of the materia medica and his ability to apply it in practice. There can be no middle ground. A man believes Homœopathy the best practice, follows it as much as he can, and is entitled to be called a homœopathic physician. A man professing to be a Christian publicly acknowledges his faith, endeavors to live up to it and therefore is entitled to be called a Christian.

Furthermore, so far as my experience goes, there is no man trying to practice Homœopathy who always succeeds. I witness the most exclusive practitioners of our art reporting cures made with the highest potencies of a drug which has never been proved, whose indications are wholly clinical. This surely is not Homœopathy. It is certainly high potency allopathy. There is, I am sorry to see, an increasing tendency, even among those who use the highest potencies, to prescribe from clinical indications and to depart from the strict law of Homœopathy. All my life I have been fighting this tendency. Those who use low potencies and crude drugs are extremely liable to neglect the study of the materia medica and to over-dose their patients. Those who use the highest potencies fill our journals with reports of cures which are allopathic. Schusslerism has infected every branch of our school. I was never more pained than when the late Dr. Hering in a way indorsed this practice. Cannot all of those who are devoted to Homœopathy and who believe that the best interests of the sick demand its perfect work unite in the advocacy of pure methods? Is it not possible so to exemplify its practice that every one will be convinced? Our enemies, the allopaths, sneer at us for our empiricism and our differences in the matter of the dose. We have a great mission. Let us not break ranks by calling each other names or imputing unworthy motives to those who follow our art.

In conclusion, I beg to make one more complaint, and that is in relation to the teaching of Homœopathy in New York. That I am incompetent to teach I freely acknowledge, but that so far as I am able to teach I must affirm Homœopathy is taught strictly and purely. The graduates of the New York College, from one chair at least, are thoroughly grounded in the best methods of practicing Homœopathy. They are taught pure symptomatology, that the best prescriptions are made from the symptoms of the patient and not from a pathological basis. They are taught the use of the single dose of any potency they may see fit to use, and they are cautioned in almost every lecture against over-dosing their patients. It must also be said that the New York College endeavors to instruct its students in all other methods in vogue in the profession. It would not have its students leave its halls ignorant of the methods pursued by other practitioners.

Besides, whatever may be said to the contrary, it is absolutely true that every physician in large practice is obliged to use other than homœopathic methods in the treatment of the sick, certainly not for their cure, but sometimes for their palliation when they cannot be cured. This practice will continue until our materia medica is so complete that every patient will be cured. Then there will be no longer any use for palliatives in any form, not even a hot-water bag or an electric battery.

Yours very truly,

T. F. Allen, M. D.

